# Comparison of the Effect of Different Local Analgesia Administration Techniques in Total Hip Arthroplasty: A Retrospective Comparative Cohort Study

**DOI:** 10.1155/2021/9914590

**Published:** 2021-07-24

**Authors:** Akira Hashimoto, Motoki Sonohata, Atsushi Kawaguchi, Sakumo Kii, Hirohito Hirata, Masaaki Mawatari

**Affiliations:** ^1^Department of Orthopaedic Surgery, Faculty of Medicine, Saga University, Nabeshima 5-1-1, Saga 849-8501, Japan; ^2^Education and Research Center for Community Medicine, Faculty of Medicine, Saga University, Nabeshima 5-1-1, Saga 849-8501, Japan

## Abstract

**Objective:**

To improve postoperative pain management, several authors have described the use of periarticular injection (PAI) or intra-articular injection (IAI) following total hip arthroplasty (THA). However, no comparative studies examining the results between PAI and IAI following THA have been published. This study aimed to evaluate the analgesic and anti-inflammatory effects of PAI and IAI following THA.

**Methods:**

This single-center, retrospective cohort study enrolled patients who underwent unilateral primary THA. A total of 278 patients (281 hips) were included in the final analyses, with 112 patients (113 hips) in the control group, 85 patients (87 hips) in the PAI group, and 81 patients (81 hips) in the IAI group. Numeric rating scale (NRS) scores and laboratory data were assessed preoperatively and on postoperative days (POD) 1 and 7.

**Results:**

NRS scores, creatine phosphokinase, and C-reactive protein levels in the PAI and IAI groups were significantly lower than those in the control group on POD 1 and 7. D-dimer levels were significantly lower in the PAI and IAI groups than in the control group on POD 7. The white blood cell count was significantly higher in the PAI and IAI groups than in the control group on POD 1 and 7. Aspartate transaminase, alanine aminotransferase, blood urea nitrogen, and creatinine levels were within the reference ranges in all three groups at all time points. NRS scores and laboratory data showed no significant differences between the PAI and IAI groups at all time points.

**Conclusion:**

PAI and IAI have equivalent analgesic and anti-inflammatory effects. Considering the technical challenges of PAI, IAI may be preferable because of its simplicity in the case of using a closed suction drain.

## 1. Introduction

Total hip arthroplasty (THA) is one of the most successful orthopedic procedures for patients with hip osteoarthritis. Adequate pain management following THA plays an important role in improving patients' overall satisfaction and enhancing functional recovery [1, 2]. Multimodal analgesia for postoperative pain following THA has gained popularity, and one aspect of multimodal analgesia is local infiltration analgesia [3–5]. Administration techniques for local infiltration analgesia can be classified into periarticular injection (PAI) and intra-articular injection (IAI) [5]. Recently, we demonstrated that PAI containing triamcinolone acetonide had analgesic and anti-inflammatory effects following THA and had the potential to accelerate early ambulation and reduce the risk of deep vein thrombosis [6]. There are several reports regarding IAI following THA [4, 7], including a comparative report between PAI and IAI following total knee arthroplasty [5]. However, to the best of our knowledge, there are no comparative studies of PAI versus IAI following THA. Additionally, PAI is relatively complicated; thus, its efficacy may differ between surgeons. In contrast, IAI via a suction drain has no technical elements. If IAI via the suction drain has analgesic and anti-inflammatory effects equivalent to PAI, it may be a simpler and more useful pain management strategy following THA.

This study aimed to investigate the effect of IAI via the suction drain and to compare IAI with PAI following THA in patients with hip osteoarthritis.

## 2. Materials and Methods

This was a single-center retrospective cohort study. The study protocol adhered to the ethical guidelines of the 1975 Declaration of Helsinki, and the study was approved by the institutional review board of our institution. All patients provided informed consent prior to participation in the study.

We implemented PAI beginning in September 2019 and IAI beginning in February 2020 at our hospital for patients undergoing THA. Thus, patients who underwent THA between May 2019 and August 2019 were included in the control group, patients who received THA between September 2019 and January 2020 comprised the PAI group, and patients who received THA between February 2020 and November 2020 comprised the IAI group. One hundred and forty patients (151 hips) were enrolled in the control group, 114 patients (137 hips) were enrolled in the PAI group, and 208 patients (221 hips) were enrolled in the IAI group. In the control group, we excluded 1 hip with hip ankylosis, 7 hips with femoral head necrosis, 5 hips with rapidly destructive coxarthrosis, 4 hips with a history of osteotomy around the hip joints, 3 hips with posttraumatic arthritis of the hip joint, 1 hip with high hip dislocation (Crowe classification [8]: type IV), 1 hip with an intraoperative fracture, 2 hips with collagen diseases, 3 hips with medical complications, and 11 hips that lacked sufficient perioperative numeric rating scale (NRS) data [9]. In the PAI group, we excluded 2 hips with hip ankylosis, 6 hips with femoral head necrosis, 2 hips with rapidly destructive coxarthrosis, 4 hips with a history of osteotomy around the hip joint, 4 hips with posttraumatic arthritis of the hip joint, 4 hips with high hip dislocation (Crowe classification [8]: type III [3 hips], type IV [1 hip]), 2 hips with collagen diseases, 8 hips of patients with diabetes, and 18 hips that lacked sufficient perioperative NRS data. In the IAI group, we excluded 7 hips with hip ankylosis, 12 hips with femoral head necrosis, 4 hips with rapidly destructive coxarthrosis, 12 hips with a history of osteotomy around the hip joint, 5 hips with posttraumatic arthritis of the hip joint, 11 hips with high hip dislocation (Crowe classification [8]: type III [2 hips], type IV [9 hips]), 7 hips with collagen diseases, 11 hips of patients with diabetes, 3 hips with Perthes disease, and 68 hips that lacked sufficient perioperative NRS data. Finally, 278 patients (281 hips) with primary hip osteoarthritis or secondary hip osteoarthritis due to developmental dysplasia of the hip joint were enrolled. Thus, the analyses included a total of 112 patients (113 hips) in the control group, 85 patients (87 hips) in the PAI group, and 81 patients (81 hips) in the IAI group (Table 1).

Anesthesia and surgery were performed according to standardized procedures. All patients received spinal anesthesia with 0.5% isobaric bupivacaine in a single shot using a 27-gauge pencil-type spinal needle at the lower lumbar level. Midazolam (2-3 mg intravenous injection) was administered for conscious sedation, if needed. In all patients, 1 g tranexamic acid was administered intravenously before the skin incision to control surgical bleeding and prevent surgical site infection. All THA procedures were performed with a cementless femoral component (PerFix-HA femoral component; Kyocera, Kyoto, Japan) and acetabular cup (AMS-HA acetabular shell; Kyocera, Kyoto, Japan) via a posterolateral approach. A closed suction drain with a porous tube (SB bag®; Sumitomo Bakelite, Tokyo, Japan) was placed into the repaired capsule, clamped for the initial 2 h, and subsequently released. The suction drain was removed 1 d after surgery. In all patients, 1 g cefazolin was intravenously administered before surgery in the operating room and three times within the time period between the patients' return to the ward and the morning after surgery.

In both the PAI and IAI groups, the regimen of local analgesics was a 41 mL solution containing 20 mL of 5 mg/mL levobupivacaine, l mL of 40 mg/mL triamcinolone acetonide (Kenacort-A® Intramuscular/Intraarticular Aqueous Suspension Injection; Bristol-Myers Squibb K.K., Tokyo, Japan), and 20 mL normal saline. In the PAI group, injections were performed after total hip prosthesis implantation and prior to closure. The surgeon injected 10 mL of the solution into the capsule, 21 mL into the gluteus and external rotators, and 10 mL into the fatty layer. In the IAI group, the solution was injected via the drain, which was inserted into the repaired capsule, after total hip prosthesis implantation and skin closure.

The postoperative analgesic protocol was the same for all groups. The patients received 50 mg flurbiprofen axetil (Ropion®; Kaken Seiyaku Co., Ltd., Tokyo, Japan) as a continuous intravenous infusion within the first 24 h after surgery (total dose = 200 mg); acetaminophen (Acelio® Intravenous Injection; Terumo Corporation, Tokyo, Japan) at 1,000 mg for patients with body weight ≥50 kg (total dose = 4000 mg) or 15 mg/kg for patients with body weight <50 kg as an intravenous infusion every 6 h during the first 24 h after surgery; and celecoxib (Celecox®; Astellas Pharma Inc., Tokyo, Japan) 200 mg orally twice daily following an initial dose of 400 mg as the standard analgesic protocol. As rescue drugs, a 50 mg diclofenac sodium suppository (Voltaren® SUPPO®; Novartis Pharma K.K., Tokyo, Japan) or 15 mg of pentazocine (intramuscular) (Sosegon® Injection; Maruishi Pharmaceutical Co., Ltd., Tokyo, Japan) were administered.

Postoperative antithrombotic therapy was the same for all groups and included the following: edoxaban (Lixiana®, Daiichi Sankyo Company, Tokyo, Japan) from the postoperative day (POD) 1 to POD 7, wearing compression stockings during the hospital stay, and early ambulation. The normal daily dose of edoxaban was 30 mg taken once orally, with some exceptions. In cases when patients weighed less than 50 kg, the creatinine (Cr) clearance was between 30 mL/min and 50 mL/min, or when the patient was ≥75 years of age, 15 mg per day was administered. Walking training within the allowable pain range was started without weight-bearing limitations, beginning 1 d after surgery.

Sex, age, body mass index (BMI), operative time, intraoperative blood loss, and postoperative blood loss were assessed. Intraoperative blood loss was calculated based on the contents of the suction bottle and the change in the weight of the surgical sponges used. Postoperative blood loss was calculated based on the drain contents.

The primary outcome was the maximum pain level assessed before surgery, on POD 1, and on POD 7. The patients' pain level was assessed using the NRS. The NRS is a segmented numeric version of the visual analog scale in which a respondent selects a whole number (integers 0–10) that best reflects the intensity of their pain. A reduction of 1.65 points in pain had been reported to demonstrate a minimal clinically important difference (MCID) for the NRS scores [10].

The secondary outcomes were the laboratory data obtained before surgery, on POD 1, and on POD 7, which were assessed using routine perioperative blood tests. Laboratory data included white blood cell count (WBC), aspartate transaminase (AST), alanine aminotransferase (ALT), creatine phosphokinase (CK), blood urea nitrogen (BUN), Cr, C-reactive protein (CRP), and D-dimer levels. Reference ranges for the laboratory data are as follows: WBC, 3300–9100/*μ*L; AST, 10–35 U/L; ALT, 5–40 U/L; CK, 40–160 U/L; BUN, 8–20 mg/dL; Cr, 0.40–0.70 mg/dL; CRP, 0.00–0.30 mg/dL; and D-dimer, 0.00–1.00 *μ*g/mL.

### 2.1. Statistical Analyses

All numerical data are expressed as mean ± standard deviation. All analyses were performed using JMP Pro software version 14.2.0 (SAS Institute Japan Ltd., Tokyo, Japan). The Shapiro–Wilk test was conducted to evaluate the distribution normality of continuous variables. Fisher's exact test was used to compare the male:female proportion among the three groups. One-way analysis of variance was used to compare the mean age among the three groups. The Kruskal–Wallis test was used to compare BMI, operative time, and intra- and postoperative blood loss among the three groups. The Steel–Dwass test was used to compare the pre- and postoperative laboratory data and the pre- and postoperative NRS scores among the three groups. Pre- and postoperative data (i.e., NRS scores and laboratory data) were compared within each group using paired *t*-test, followed by Bonferroni correction. Standard least-squares regression was employed to estimate the contribution of dependent variables to the NRS scores and blood sample.

## 3. Results and Discussion

### 3.1. Results

No significant differences in age, sex, BMI, operative time, and intra- or postoperative blood loss were observed among the three groups (Table 1).

**Figure 1:**
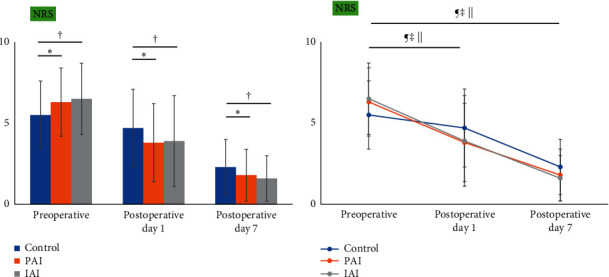
Comparison of NRS scores among the three groups (a) and within each group (b). Significant differences between the control and PAI groups are marked as ^*∗*^*p* < 0.05. Significant differences between the control and IAI groups are marked as ^†^*p* < 0.05. Significant differences between the PAI and IAI groups are marked as ^§^*p* < 0.05. Significant differences in perioperative data within the control group are marked as ^¶^*p* < 0.05. Significant differences in perioperative data within the PAI group are marked as ^‡^*p* < 0.05. Significant differences in perioperative data within the IAI group are marked as ^||^*p* < 0.05. PAI, periarticular injection; IAI, intra-articular injection; NRS, numeric rating scale.

The preoperative NRS scores of the PAI and IAI groups were significantly higher than those of the control group, whereas the postoperative NRS scores of the PAI and IAI groups were significantly lower than those of the control group (Table 2, Figure 1(a)). Pre- and postoperative NRS scores showed no significant differences between the PAI and IAI groups (Table 2, Figure 1(a)). The proportion of patients in the control, PAI, and IAI groups who achieved the MCID on POD 1 was 35.4%, 71.3%, and 61.7%, respectively, whereas the proportion of patients in the control, PAI, and IAI groups who achieved the MCID on POD 7 was 78.8%, 88.5%, and 91.4%, respectively (Table 3). A positive linear relationship between preoperative and postoperative NRS scores was identified (Supplementary Table 1).

**Figure 2:**
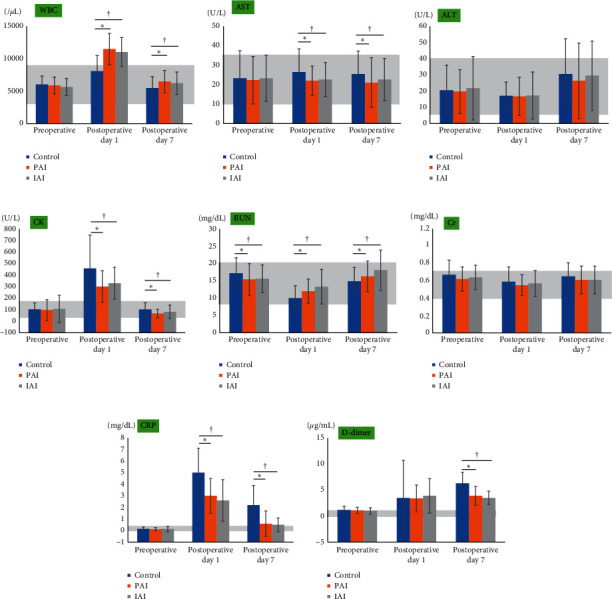
Comparison of laboratory data among the three groups (a–h). The gray area represents the reference range for each laboratory value. Significant differences between the control and PAI groups are marked as ^*∗*^*p* < 0.05. Significant differences between the control and IAI groups are marked as ^†^*p* < 0.05. Significant differences between the PAI and IAI groups are marked as ^§^*p* < 0.05. PAI, periarticular injection; IAI, intra-articular injection; WBC, white blood cell count; AST, aspartate transaminase; ALT, alanine aminotransferase; CK, creatine phosphokinase; BUN, blood urea nitrogen; Cr, creatinine; CRP, C-reactive protein.

**Figure 3:**
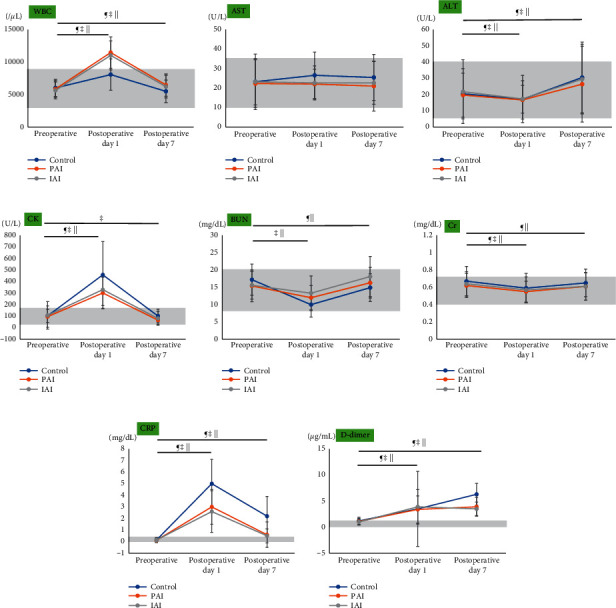
Comparison of laboratory data within each group (a–h). The gray area represents the reference range for each laboratory value. Significant differences in perioperative data within the control group are marked as ^¶^*p* < 0.05. Significant differences in perioperative data within the PAI group are marked as ^‡^*p* < 0.05. Significant differences in perioperative data within the IAI group are marked as ^||^*p* < 0.05. PAI, periarticular injection; IAI, intra-articular injection; WBC, white blood cell count; AST, aspartate transaminase; ALT, alanine aminotransferase; CK, creatine phosphokinase; BUN, blood urea nitrogen; Cr, creatinine; CRP, C-reactive protein.

Tables 4–6 and Figures 2 and 3 present a comparison of laboratory values among the three groups and a comparison of perioperative laboratory data within each group. Preoperative WBC showed no significant difference among the three groups (Table 4, Figure 2(a)). The WBC was significantly higher in the PAI and IAI groups than in the control group on POD 1 and POD 7, with no significant differences in WBC between the PAI and IAI groups (Tables 5 and 6, Figure 2(a)). Within the PAI and IAI groups, the WBC was significantly higher on POD 7 than preoperatively (Figure 3(a)). Preoperative CK levels showed no significant differences among the three groups (Table 4, Figure 2(d)). The CK levels were significantly lower in the PAI and IAI groups than in the control group on POD 1 and POD 7, with no significant differences in CK levels between the PAI and IAI groups (Tables 5 and 6, Figure 2(d)). Preoperative CRP levels showed no significant differences among the three groups (Table 4, Figure 2(g)). The CRP levels were lower in the PAI and IAI groups than in the control group on POD 1 and POD 7, with no significant differences in CRP levels between the PAI and IAI groups (Tables 5 and 6, Figure 2(g)). The D-dimer levels showed no significant differences among the three groups preoperatively and on POD 1 (Tables 4 and 5, Figure 2(h)). The D-dimer levels were significantly lower in the PAI and IAI groups than in the control group on POD 7, with no significant differences in D-dimer levels between the PAI and IAI groups (Table 6, Figure 2(h)). The AST, ALT, BUN, and Cr levels were within the reference ranges for all three groups preoperatively and on POD 1 and POD 7 (Tables 4–6; Figures 2(b)–2(f) and 3(b)–3(f)). The AST levels were lower in the PAI and IAI groups than in the control group on POD 1 and POD 7, with no significant differences in AST levels between the PAI and IAI groups (Tables 5 and 6, Figure 2(b)). The preoperative BUN levels were significantly lower in the PAI and IAI groups than in the control group; conversely, the postoperative BUN levels were significantly higher in the PAI and IAI groups than in the control group (Tables 4–6, Figure 2(e)).

### 3.2. Discussion

To the best of our knowledge, this study is the first to investigate the analgesic and anti-inflammatory effects of PAI versus those of IAI following THA. Our findings indicate that PAI and IAI containing triamcinolone acetonide have equivalent analgesic and anti-inflammatory effects.

Levobupivacaine, which is the S-enantiomer of bupivacaine, is a long-acting local anesthetic drug [11]. However, previous studies have found that PAI (levobupivacaine and/or epinephrine) during THA did not reduce postoperative pain [12, 13]. Triamcinolone acetonide is an intermediate-acting glucocorticoid that provides a slow absorption time and prolonged duration when administered intramuscularly [14–16]. Glucocorticoids have an anti-inflammatory effect by inhibiting the synthesis of phospholipase A2 [14, 17]. The anti-inflammatory effect of glucocorticoids results in reduced postoperative CRP levels [6, 18]. In this study, postoperative CRP levels in the PAI and IAI groups were significantly lower than those in the control group, and there was no significant difference between the PAI and IAI groups. CK is primarily found in muscle tissues, and CK elevation is a feature of muscle inflammation or damage [19, 20]. As AST is found in other organs, it is not a highly specific marker of muscle damage [21]. Nonetheless, AST is a leakage enzyme commonly used in detecting muscle damage [22]. In this study, postoperative AST and CK levels in the PAI and IAI groups were significantly lower than those in the control group, with no significant differences between the PAI and IAI groups. Therefore, triamcinolone acetonide may play an important anti-inflammatory role after THA. Considering the postoperative CRP, AST, and CK levels, IAI and PAI have equivalent anti-inflammatory effects, which last until at least POD 7. Postoperative pain is caused by an inflammatory reaction and the initiation of an afferent neuronal response following surgical invasion [23]. Hence, reducing inflammation is important for reducing postoperative pain. In this study, postoperative NRS scores in the PAI and IAI groups were significantly lower than those in the control group, and there was no significant difference between the PAI and IAI groups. Therefore, IAI and PAI have equivalent analgesic effects, which last until at least POD 7.

Postoperative management of inflammation and pain following THA is important for early postoperative rehabilitation [24, 25]. Early postoperative rehabilitation can help prevent deep vein thrombosis in patients after surgery [26, 27]. A D-dimer test is one of the methods for diagnosing deep vein thrombosis [26]. In this study, D-dimer levels on POD 7 in the PAI and IAI groups were significantly lower than those in the control group, and there was no significant difference between the PAI and IAI groups. Considering D-dimer levels at POD 7 in the PAI and IAI groups, the anti-inflammatory and analgesic effects of PAI and IAI may have equivalent potential to accelerate early ambulation and reduce the risk of deep vein thrombosis [6].

Glucocorticoids increase the number of circulating neutrophils by stimulating the bone marrow to produce more granulocytes, inhibiting neutrophil apoptosis, and impairing the migration of granulocytes to sites of inflammation or infection through the vasculature [28–30]. In this study, the postoperative WBC levels in the PAI and IAI groups were significantly higher than those in the control group, with no significant differences between the PAI and IAI groups. In the PAI and IAI groups, the WBC levels on POD 7 were significantly higher than they were before surgery. This indicates that the effect on granulocytes also lasted until at least POD 7 and that the effect was equivalent in the PAI and IAI groups, as was the anti-inflammatory effect of glucocorticoids.

BUN is a nitrogenous end product of protein metabolism [31]. Glucocorticoids inhibit protein synthesis and stimulate protein degradation in skeletal muscles [32]. In the present study, the BUN levels were higher postoperatively in the PAI and IAI groups than in the control group; however, the BUN levels in the PAI and IAI groups were lower on POD 1 than before surgery. Therefore, the glucocorticoids used in this study might not have affected the postoperative BUN levels.

AST and ALT have been regarded as markers of liver injury [33]. Levobupivacaine and corticosteroids are mainly metabolized by the liver [34, 35]. In this study, postoperative AST and ALT levels were within their reference ranges in both the PAI and IAI groups. Therefore, there was no drug hepatopathy following THA in either analgesic group. BUN and Cr are biomarkers of kidney function [36]. In this study, postoperative BUN and Cr levels were within their reference ranges in both the PAI and IAI groups. Therefore, there was no drug-induced liver or kidney injury following THA in either the PAI or IAI group.

In a study examining the innervation of the soft tissue in the human hip joint, innervation was greater in the muscle and superficial fasciae than in the tendon and capsule [37]. This may indicate that local infiltration analgesia of the muscle and fasciae is important for pain management following THA. In contrast, in the IAI group, levobupivacaine containing triamcinolone acetonide was retrogradely injected via the drain, which was inserted into the capsule; there was no direct injection into the muscle; however, IAI had equivalent analgesic and anti-inflammatory effects compared with PAI. Drain clamping and/or topical administration of tranexamic acid are often used to reduce postoperative blood loss following THA [38]. In this study, the suction drain was routinely clamped for the initial 2 h to reduce postoperative blood loss. A previous study utilizing IAI via the drain during total knee arthroplasty also employed drain clamping for the infiltration of analgesics and found an analgesic effect with IAI [39]. Postoperative blood loss was calculated by measuring the contents of the drain, and there were no significant differences among the three groups in our study, even though 10 mL of analgesic solution was injected into the capsule in the PAI group and 41 mL of analgesic solution was injected into the capsule in the IAI group. Therefore, in this study, the analgesic solution injected into the capsule in the PAI and IAI groups may have infiltrated the areas around the hip joint during drain clamping. It is assumed that during IAI, the infiltrated analgesic solution spread to the gluteus, external rotators, and fascia, resulting in anti-inflammatory and analgesic effects equivalent to those observed with PAI.

A closed suction drain has been widely used after THA to reduce the chance of hematoma formation and eliminate this potential risk of infection [40]. However, recent studies showed no clinical benefit to reducing hematoma and infection rates, the disadvantage of a higher transfusion rate, and a longer postoperative length of hospital stay when using a closed suction drain [41, 42]. Therefore, the use of a closed suction drain in THA remains controversial in terms of the benefit to outcome in THA [41]. In the present study, PAI and IAI were effective treatments for pain and inflammation following THA. Hence, IAI is a simpler method than PAI in the case of using a closed suction drain, considering the technical aspects of PAI. However, PAI may also be an effective method regardless of the use of a closed suction drain.

This study has several limitations. First, this was a retrospective study with a relatively high rate of missing data in the IAI group. Therefore, a high-quality randomized controlled trial is needed in the future. Second, we did not investigate the association between postoperative functional performance and D-dimer levels. In the future, an assessment of postoperative functional performance is needed. Third, the incidence of DVT was not assessed. Although imaging tests are not always necessary for the diagnosis of DVT [26], imaging tests may be needed to determine whether PAI and IAI containing a corticosteroid accurately reduce DVT in future studies.

## 4. Conclusions

In conclusion, PAI and IAI have equivalent analgesic and anti-inflammatory effects. Considering the technical aspects of PAI, IAI may be a simpler, and therefore preferable, method in the case of using a closed suction drain.

## Figures and Tables

**Table 1:** Demographic data of the IAI, PAI, and control groups.

	Control group	PAI group	IAI group	*p* value
Number of patients (hips)	112 (113)	85 (87)	81 (81)	
Females, *n* (%)	95 (85)	77 (91)	72 (89)	0.5533
Age (years)	65.3 ± 8.8	65.9 ± 10.3	66.0 ± 10.3	0.8767
BMI (kg/m^2^)	24.1 ± 3.2	24.2 ± 4.9	24.2 ± 5.0	0.8742
Operative time (min)	47.8 ± 9.0	51.1 ± 19.9	47.5 ± 7.8	0.7198
Intraoperative blood loss (g)	244.3 ± 90.0	248.6 ± 119.1	224.2 ± 88.0	0.1350
Postoperative blood loss (g)	164.3 ± 133.1	149.0 ± 98.5	133.1 ± 99.7	0.2283

Values are expressed as mean ± standard deviation. PAI, periarticular injection; IAI, intra-articular injection; BMI, body mass index.

**Table 2:** Comparison of NRS scores among the three groups.

	Control group (*N* = 113)	PAI group (*N* = 87)	IAI group (*N* = 81)	*p* value	95% confidence interval
NRS scores					
Preoperative	5.5 ± 2.1	6.3 ± 2.1	6.5 ± 2.2	C versus P: 0.0110	0–2.0
				C versus I: 0.0024	0–2.0
				P versus I: 0.7757	−1.0–1.0
POD 1	4.7 ± 2.4	3.8 ± 2.4	3.9 ± 2.8	C versus P: 0.0142	−2.0–0
				C versus I: 0.0444	−2.0–0
				P versus I: 0.9939	−1.0–1.0
POD 7	2.3 ± 1.7	1.8 ± 1.6	1.6 ± 1.4	C versus P: 0.0278	−1.0–0
				C versus I: 0.0322	−1.0–0
				P versus I: 0.9985	−1.0–1.0

Values are expressed as mean ± standard deviation. NRS, numeric rating scale; PAI and P, periarticular injection; IAI and I, intra-articular injection; C, control; POD, postoperative.

**Table 3:** MCID for the NRS scores of the three groups.

	POD 1	POD 7
Control group (*N* = 113)		
MCID (points)	0.7 ± 2.8 (0.2–1.2)	3.2 ± 2.3 (2.8–3.7)
Improvement of >1.65 points, *n* (%)	40 (35.4)	89 (78.8)

PAI group (*N* = 87)		
MCID (points)	2.5 ± 2.7 (1.9–3.1)	4.5 ± 2.4 (4.0–5.0)
Improvement of >1.65 points, *n* (%)	62 (71.3)	77 (88.5)

IAI group (*n* = 81)		
MCID (points)	2.6 ± 3.3 (1.9–3.4)	4.9 ± 2.4 (4.4–5.4)
Improvement of >1.65 points, *n* (%)	50 (61.7)	74 (91.4)

Numerical data are expressed as mean ± standard deviation (95% confidence interval). MCID, minimal clinically important difference; NRS, numeric rating scale; POD, postoperative; PAI, periarticular injection; IAI, intra-articular injection.

**Table 4:** Comparison of preoperative laboratory data among the three groups.

Laboratory data	Preoperative				
	Control group	PAI group	IAI group	*p* value	95% confidence interval
	(*N* = 113)	(*N* = 87)	(*N* = 81)		
WBC (/*μ*L)	6019.4 ± 1653.6	5851.7 ± 1299.5	5639.5 ± 1308.7	C versus P: 0.9420	−500.0–400.0
				C versus I: 0.4403	−800.0–300.0
				P versus I: 0.7511	−700.0–300.0

AST (U/L)	23.3 ± 14.2	22.3 ± 12.2	23.2 ± 11.9	C versus P: 0.1170	−3.0–0
				C versus I: 0.5168	−3.0–1.0
				P versus I: 0.6517	−1.0–2.0

ALT (U/L)	20.5 ± 15.5	19.7 ± 13.5	21.8 ± 19.6	C versus P: 0.5483	−3.0–1.0
				C versus I: 0.8612	−3.0–2.0
				P versus I: 0.9028	−2.0–3.0

CK (U/L)	102.6 ± 56.4	96.1 ± 92.2	107.3 ± 118.9	C versus P: 0.1407	−22.0–3.0
				C versus I: 0.8648	−17.0–10.0
				P versus I: 0.4645	−6.0–20.0

BUN (mg/dL)	17.2 ± 4.5	15.4 ± 4.6	15.6 ± 4.0	C versus P: 0.0138	−3.1–0.3
				C versus I: 0.0320	−2.9–0.1
				P versus I: 0.9344	−1.2–1.6

Cr (mg/dL)	0.67 ± 0.17	0.62 ± 0.14	0.64 ± 0.14	C versus P: 0.1635	−0.08–0.01
				C versus I: 0.7456	−0.06–0.03
				P versus I: 0.5656	−0.03–0.07

CRP (mg/dL)	0.14 ± 0.17	0.12 ± 0.15	0.14 ± 0.22	C versus P: 0.9598	−0.03–0.02
				C versus I: 0.9631	−0.03–0.02
				P versus I: 0.9939	−0.02–0.03

D-dimer (*μ*g/mL)	1.2 ± 0.7	1.1 ± 0.6	1.0 ± 0.6	C versus P: 0.6273	−0.16–0.07
				C versus I: 0.3123	−0.2–0.05
				P versus I: 0.8705	−0.15–0.1

Values are expressed as mean ± standard deviation. PAI and P, periarticular injection; IAI and I, intra-articular injection; C, control; WBC, white blood cell count; AST, aspartate transaminase; ALT, alanine aminotransferase; CK, creatine phosphokinase; BUN, blood urea nitrogen; Cr, creatinine; CRP, C-reactive protein.

**Table 5:** Comparison of laboratory data on POD 1 among the three groups.

Laboratory data	POD 1				
	Control group	PAI group	IAI group	*p* value	95% confidence interval
	(*N* = 113)	(*N* = 87)	(*N* = 81)		
WBC (/*μ*L)	8072.6 ± 1654.2	11470.1 ± 2409.0	11025.9 ± 2220.9	C versus P: <0.0001	2600.0–4100.0
				C versus I: <0.0001	2200.0–3700.0
				P versus I: 0.4925	−1300.0–500.0

AST (U/L)	26.5 ± 11.9	22.0 ± 7.6	22.6 ± 8.7	C versus P: <0.0001	−6.0–2.0
				C versus I: 0.0008	−5.0–1.0
				P versus I: 0.4869	−1.0–3.0

ALT (U/L)	17.1 ± 8.5	16.7 ± 11.8	17.3 ± 14.5	C versus P: 0.1556	−3.0–0
				C versus I: 0.4459	−3.0–1.0
				P versus I: 0.7745	−1.0–2.0

CK (U/L)	457.8 ± 289.2	301.1 ± 136.7	329.5 ± 138.3	C versus P: <0.0001	−172.0–65.0
				C versus I: 0.0002	−146.0–39.0
				P versus I: 0.3262	−18.0–73.0

BUN (mg/dL)	10.0 ± 3.6	12.0 ± 3.5	13.3 ± 5.0	C versus P: <0.0001	1.0–2.9
				C versus I: <0.0001	1.7–3.9
				P versus I: 0.2429	−0.4–2.0

Cr (mg/dL)	0.59 ± 0.17	0.55 ± 0.12	0.57 ± 0.15	C versus P: 0.1745	−0.07–0.01
				C versus I: 0.3808	−0.06–0.02
				P versus I: 0.8890	−0.03–0.05

CRP (mg/dL)	5.0 ± 2.1	3.0 ± 1.5	2.6 ± 1.8	C versus P: <0.0001	−2.5–1.3
				C versus I: <0.0001	−3.0–1.74
				P versus I: 0.0921	−1.04–0.06

D-dimer (*μ*g/mL)	3.5 ± 7.2	3.4 ± 2.6	3.9 ± 3.3	C versus P: 0.8024	−0.6–0.36
				C versus I: 0.9685	−3.0–1.74
				P versus I: 0.7607	−1.04–0.06

Values are expressed as mean ± standard deviation. PAI and P, periarticular injection; IAI and I, intra-articular injection; C, control; POD, postoperative day; WBC, white blood cell count; AST, aspartate transaminase; ALT, alanine aminotransferase; CK, creatine phosphokinase; BUN, blood urea nitrogen; Cr, creatinine; CRP, C-reactive protein.

**Table 6:** Comparison of laboratory data on POD 7 among the three groups.

Laboratory data	POD 7				
	Control group	PAI group	IAI group	*p* value	95% confidence interval
	(*N* = 113)	(*N* = 87)	(*N* = 81)		
WBC (/*μ*L)	5491.2 ± 1274.9	6464.4 ± 1726.4	6221.0 ± 1737.5	C versus P: <0.0001	400.0–1400.0
				C versus I: 0.0011	300.0–1200.0
				P versus I: 0.7549	−800.0–400.0

AST (U/L)	25.4 ± 11.8	21 ± 12.8	22.6 ± 10.9	C versus P: <0.0001	−6.0–2.0
				C versus I: 0.0206	−4.0–0
				P versus I: 0.0647	0–4.0

ALT (U/L)	30.5 ± 21.8	26.4 ± 23.3	29.5 ± 21.4	C versus P: 0.1795	−6.0–1.0
				C versus I: 0.9966	−4.0–4.0
				P versus I: 0.1624	−1.0–6.0

CK (U/L)	101.2 ± 59.0	66.0 ± 36.8	80.8 ± 59.5	C versus P: <0.0001	−42.0–15.0
				C versus I: 0.0042	−33.0–5.0
				P versus I: 0.1552	−2.0–20.0

BUN (mg/dL)	14.9 ± 4.0	16.3 ± 4.4	18.1 ± 5.8	C versus P: 0.0247	0.1–2.7
				C versus I: <0.0001	1.3–3.9
				P versus I: 0.1161	−0.2–2.7

Cr (mg/dL)	0.65 ± 0.16	0.61 ± 0.16	0.61 ± 0.16	C versus P: 0.0719	−0.08–0
				C versus I: 0.2520	−0.07–0.01
				P versus I: 0.9540	−0.04–0.05

CRP (mg/dL)	2.2 ± 1.7	0.6 ± 1.1	0.5 ± 0.6	C versus P: <0.0001	−1.58–1.06
				C versus I: <0.0001	−1.62–1.11
				P versus I: 0.8720	−0.11–0.07

D-dimer (*μ*g/mL)	6.3 ± 2.1	3.9 ± 1.8	3.5 ± 1.3	C versus P: <0.0001	−2.88–1.78
				C versus I: <0.0001	−3.13–2.06
				P versus I: 0.4233	−0.76–0.23

Values are expressed as mean ± standard deviation. PAI and P, periarticular injection; IAI and I, intra-articular injection; C, control; POD, postoperative day; WBC, white blood cell count; AST, aspartate transaminase; ALT, alanine aminotransferase; CK, creatine phosphokinase; BUN, blood urea nitrogen; Cr, creatinine; CRP, C-reactive protein.

## Data Availability

The data used to support the findings of this study are available from the corresponding author upon request.

## Supplementary Materials

Supplementary Materials Estimation of the contribution of dependent variables to the NRS scores and blood sample.
