# Supplementary material for: Comparison of the Effect of Different Local Analgesia Administration Techniques in Total Hip Arthroplasty: A Retrospective Comparative Cohort Study
Source: Pain Res Manag. 2021 Jul 24;2021:9914590. doi: 10.1155/2021/9914590 (PMC8328737; doi:10.1155/2021/9914590)
Supplement: Supplementary Materials — Estimation of the contribution of dependent variables to the NRS scores and blood sample. [file 9914590.f1.docx]

**Supplementary Materials**

**Supplementary Table 1**

Contribution to the change in NRS scores from before surgery to POD 1 and POD 7

|  | Dependent variables | Estimate | Standard error | *T* ratio | *p*-value |
| --- | --- | --- | --- | --- | --- |
| Change from before surgery to POD 1 | |  |  |  |  |
| Control group (*N*=113) | |  |  |  |  |
|  | Intercept | -4.3705 | 2.1923 | -1.99 | 0.0487* |
|  | Preoperative NRS scores | 0.7036 | 0.1073 | 6.56 | <0.0001* |
|  | Sex (female) | -0.4627 | 0.3016 | -1.53 | 0.1279 |
|  | Age | 0.0423 | 0.0216 | 1.96 | 0.0526 |
|  | BMI | -0.0503 | 0.0689 | -0.73 | 0.4668 |
| PAI group (*N*=87) | |  |  |  |  |
|  | Intercept | -4.5875 | 2.5024 | -1.83 | 0.0704 |
|  | Preoperative NRS scores | 0.6780 | 0.1210 | 5.60 | <0.0001* |
|  | Sex (female) | -0.2541 | 0.3892 | -0.65 | 0.5157 |
|  | Age | 0.0240 | 0.0285 | 0.84 | 0.4013 |
|  | BMI | 0.0602 | 0.0513 | 1.17 | 0.2441 |
| IAI group (*N*=81) | |  |  |  |  |
|  | Intercept | -4.8710 | 2.6780 | -1.82 | 0.0729 |
|  | Preoperative NRS scores | 0.7904 | 0.1450 | 5.45 | <0.0001* |
|  | Sex (female) | -0.3939 | 0.5143 | -0.77 | 0.4461 |
|  | Age | 0.0451 | 0.3010 | 1.45 | 0.1501 |
|  | BMI | -0.0136 | 0.0645 | -0.21 | 0.8339 |
| Change from before surgery to POD 7 | |  |  |  |  |
| Control group (*N*=113) | |  |  |  |  |
|  | Intercept | 0.6010 | 1.5468 | 0.39 | 0.6984 |
|  | Preoperative NRS scores | 0.8863 | 0.0757 | 11.70 | <0.0001* |
|  | Sex (female) | -0.0228 | 0.0152 | -1.50 | 0.1374 |
|  | Age | 0.0486 | 0.2128 | 0.23 | 0.8196 |
|  | BMI | -0.0321 | 0.0487 | -0.66 | 0.5115 |
| PAI group (*N*=87) | |  |  |  |  |
|  | Intercept | -1.1724 | 1.7557 | -0.67 | 0.5061 |
|  | Preoperative NRS scores | 0.8916 | 0.0849 | 10.50 | <0.0001* |
|  | Sex (female) | -0.2966 | 0.2730 | 0.22 | 0.8265 |
|  | Age | 0.0044 | 0.0200 | -1.09 | 0.2806 |
|  | BMI | 0.0006 | 0.0360 | 0.02 | 0.9872 |
| IAI group (*N*=81) | |  |  |  |  |
|  | Intercept | -1.2308 | 1.3056 | -0.94 | 0.3488 |
|  | Preoperative NRS scores | 0.9076 | 0.0707 | 12.84 | <0.0001* |
|  | Sex (female) | -0.0471 | 0.2507 | -0.19 | 0.8496 |
|  | Age | 0.0076 | 0.0151 | 0.51 | 0.6143 |
|  | BMI | -0.0114 | 0.0315 | -0.36 | 0.7189 |

* indicates significant difference at *p*<0.05. NRS, numeric rating scale; POD, postoperative day; BMI, body mass index; PAI, peri-articular injection; IAI, intra-articular injection.

**Supplementary Table 2**

Contribution to the change in WBC from before surgery to POD 1 and POD 7.

|  | Dependent variables | | Estimate | Standard error | *T* ratio | *p*-value |
| --- | --- | --- | --- | --- | --- | --- |
| Change from before surgery to POD 1 | | |  |  |  |  |
| Control group (*N*=113) | | |  |  |  |  |
|  | Intercept | | 3446.6687 | 1429.364 | 2.41 | 0.0176* |
|  | Preoperative NRS scores | | -108.1392 | 69.9767 | -1.55 | 0.1252 |
|  | Sex (female) | | 89.5944 | 196.6095 | -0.46 | 0.6495 |
|  | Age | | -7.9973 | 14.0950 | -0.57 | 0.5716 |
|  | BMI | | -14.2641 | 44.9699 | -0.32 | 0.7517 |
| PAI group (*N*=87) | | |  |  |  |  |
|  | Intercept | | 8719.9233 | 2238.1450 | 3.90 | 0.0002* |
|  | Preoperative NRS scores | | -144.3964 | 108.2196 | -1.33 | 0.1858 |
|  | Sex (female) | | -352.4953 | 348.0720 | -1.01 | 0.3142 |
|  | Age | | -30.3327 | 25.4637 | -1.19 | 0.2370 |
|  | BMI | | 2.4485 | 45.9366 | 0.05 | 0.9576 |
| IAI group (*N*=81) | | |  |  |  |  |
|  | Intercept | | 6916.6285 | 1665.324 | 4.15 | <0.0001* |
|  | Preoperative NRS scores | | -26.2435 | 89.9759 | -0.29 | 0.7713 |
|  | Sex (female) | | 83.7492 | 318.6176 | 0.26 | 0.7934 |
|  | Age | | -17.5017 | 19.3264 | -0.91 | 0.3681 |
|  | BMI | | -11.2125 | 40.0201 | -0.28 | 0.7801 |
| Change from before surgery to POD 7 | | |  |  |  |  |
| Control group (*N*=113) | |  | |  |  |  |
|  | Intercept | -1008.6450 | | 1180.084 | -0.85 | 0.3946 |
|  | Preoperative NRS scores | -34.65600 | | 57.7728 | -0.60 | 0.5499 |
|  | Sex (female) | -121.4386 | | 162.3210 | -0.75 | 0.4560 |
|  | Age | 10.0354 | | 11.6369 | 0.86 | 0.3904 |
|  | BMI | 3.6869 | | 37.1272 | 0.10 | 0.9211 |
| PAI group (*N*=87) | |  | |  |  |  |
|  | Intercept | -1599.8068 | | 1306.9670 | 1.22 | 0.2244 |
|  | Preoperative NRS scores | -3.3379 | | 63.1920 | -0.05 | 0.9580 |
|  | Sex (female) | -208.8064 | | 203.2570 | -1.03 | 0.3073 |
|  | Age | -18.8818 | | 14.8695 | -1.27 | 0.2077 |
|  | BMI | 17.7062 | | 26.8247 | 0.66 | 0.5111 |
| IAI group (*N*=81) | |  | |  |  |  |
|  | Intercept | 1740.9443 | | 1341.433 | 1.30 | 0.1983 |
|  | Preoperative NRS scores | -71.0119 | | 72.6099 | -0.98 | 0.3312 |
|  | Sex (female) | -405.1015 | | 257.6130 | -1.57 | 0.1200 |
|  | Age | -4.6649 | | 15.5224 | -0.30 | 0.7646 |
|  | BMI | -2.96212 | | 32.3147 | -0.09 | 0.9272 |

* indicates significant difference at *p*<0.05. WBC, white blood cell count; POD, postoperative day; NRS, numeric rating scale; BMI, body mass index; PAI, peri-articular injection; IAI, intra-articular injection.

**Supplementary Table 3**

Contribution to the change in AST from before surgery to POD 1 and POD 7.

|  | Dependent variables | Estimate | Standard error | *T* ratio | *p*-value |
| --- | --- | --- | --- | --- | --- |
| Change from before surgery to POD 1 | |  |  |  |  |
| Control group (*N*=113) | |  |  |  |  |
|  | Intercept | 3.6629 | 15.4967 | 0.24 | 0.8136 |
|  | Preoperative NRS scores | 0..1926 | 0.7587 | 0.25 | 0.8001 |
|  | Sex (female) | 0.7481 | 2.1316 | 0.35 | 0.7263 |
|  | Age | -0.0683 | 0.1528 | -0.45 | 0.6558 |
|  | BMI | 0.1052 | 0.4875 | 0.22 | 0.8295 |
| PAI group (*N*=87) | |  |  |  |  |
|  | Intercept | -5.7210 | 8.6320 | -0.66 | 0.5093 |
|  | Preoperative NRS scores | 0.0878 | 0.4173 | 0.21 | 0.8338 |
|  | Sex (female) | -0.0499 | 1.3424 | -0.04 | 0.9704 |
|  | Age | 0.0801 | 0.0982 | 0.82 | 0.4170 |
|  | BMI | -0.0139 | 0.1772 | -0.08 | 0.9377 |
| IAI group (*N*=81) | |  |  |  |  |
|  | Intercept | 2.5300 | 6.3602 | 0.40 | 0.6919 |
|  | Preoperative NRS scores | -0.3306 | 0.3443 | -0.96 | 0.3399 |
|  | Sex (female) | -0.6608 | 1.2214 | -0.54 | 0.5901 |
|  | Age | 0.0592 | 0.0736 | 0.80 | 0.4240 |
|  | BMI | -0.1815 | 0.1532 | -1.18 | 0.2397 |
| Change from before surgery to POD 7 | |  |  |  |  |
| Control group (*N*=113) | |  |  |  |  |
|  | Intercept | 22.5199 | 16.1085 | 1.40 | 0.1650 |
|  | Preoperative NRS scores | 0.8858 | 0.7886 | 1.12 | 0.2638 |
|  | Sex (female) | -1.3701 | 2.2157 | -0.62 | 0.5376 |
|  | Age | -0.1685 | 0.1588 | -1.06 | 0.2912 |
|  | BMI | -0.5469 | 0.5068 | -1.08 | 0.2829 |
| PAI group (*N*=87) | |  |  |  |  |
|  | Intercept | 14.4302 | 15.2991 | 0.94 | 0.3483 |
|  | Preoperative NRS scores | 0.1567 | 0.7397 | 0.21 | 0.8327 |
|  | Sex (female) | -2.5959 | 2.3793 | -1.09 | 0.2784 |
|  | Age | -0.0858 | 0.1741 | -0.49 | 0.6234 |
|  | BMI | -0.3759 | 0.3140 | -1.20 | 0.2347 |
| IAI group (*N*=81) | |  |  |  |  |
|  | Intercept | 16.2736 | 9.2959 | 1.75 | 0.0840 |
|  | Preoperative NRS scores | -0.1707 | 0.5031 | -0.34 | 0.7354 |
|  | Sex (female) | -0.3413 | 1.7852 | -0.19 | 0.8489 |
|  | Age | -0.1165 | 0.1075 | -1.08 | 0.2821 |
|  | BMI | -0.3210 | 0.2239 | -1.43 | 0.1558 |

* indicates significant difference at *p*<0.05. AST, aspartate transaminase; POD, postoperative day; NRS, numeric rating scale; BMI, body mass index; PAI, peri-articular injection; IAI, intra-articular injection.

**Supplementary Table 4**

Contribution to the change in ALT from before surgery to POD 1 and POD 7.

|  | Dependent variables | Estimate | Standard error | *T* ratio | *p*-value |
| --- | --- | --- | --- | --- | --- |
| Change from before surgery to POD 1 | |  |  |  |  |
| Control group (*N*=113) | |  |  |  |  |
|  | Intercept | -5.4036 | 10.8785 | -0.50 | 0.6204 |
|  | Preoperative NRS scores | 0.4335 | 0.5326 | 0.81 | 0.4175 |
|  | Sex (female) | 1.3559 | 1.4963 | 0.91 | 0.3669 |
|  | Age | -0.0194 | 0.1073 | -0.18 | 0.8569 |
|  | BMI | 0.0034 | 0.3423 | 0.01 | 0.9922 |
| PAI group (*N*=87) | |  |  |  |  |
|  | Intercept | -13.6839 | 5.9815 | -2.29 | 0.0247* |
|  | Preoperative NRS scores | 0.7646 | 0.2892 | 2.64 | 0.0098* |
|  | Sex (female) | 1.2780 | 0.9302 | 1.37 | 0.1732 |
|  | Age | 0.1429 | 0.0681 | 2.10 | 0.0388* |
|  | BMI | -0.1849 | 0.1228 | -1.50 | 0.1363 |
| IAI group (*N*=81) | |  |  |  |  |
|  | Intercept | -6.0493 | 7.2729 | -0.83 | 0.4082 |
|  | Preoperative NRS scores | 0.0163 | 0.3937 | 0.04 | 0.9670 |
|  | Sex (female) | 0.1596 | 1.3967 | 0.11 | 0.9093 |
|  | Age | 0.1425 | 0.0842 | 1.69 | 0.0944 |
|  | BMI | -0.3361 | 0.1752 | -1.92 | 0.0588 |
| Change from before surgery to POD 7 | |  |  |  |  |
| Control group (*N*=113) | |  |  |  |  |
|  | Intercept | 42.4594 | 24.1763 | 1.76 | 0.0819 |
|  | Preoperative NRS scores | 1.9613 | 1.1836 | 1.66 | 0.1004 |
|  | Sex (female) | 1.0606 | 3.3254 | 0.32 | 0.7504 |
|  | Age | -0.4109 | 0.2384 | -1.72 | 0.0877 |
|  | BMI | -0.6915 | 0.7606 | -0.91 | 0.3653 |
| PAI group (*N*=87) | |  |  |  |  |
|  | Intercept | 32.7764 | 23.2349 | 1.41 | 0.1621 |
|  | Preoperative NRS scores | 0.5362 | 1.1235 | 0.48 | 0.6344 |
|  | Sex (female) | -6.6916 | 3.6134 | -1.85 | 0.0676 |
|  | Age | -0.0996 | 0.2643 | -0.38 | 0.7074 |
|  | BMI | -0.7345 | 0.47682 | -1.54 | 0.1274 |
| IAI group (*N*=81) | |  |  |  |  |
|  | Intercept | 14.1264 | 15.6694 | 0.90 | 0.3702 |
|  | Preoperative NRS scores | 0.3261 | 0.8482 | 0.38 | 0.7017 |
|  | Sex (female) | -1.2256 | 3.0009 | -0.41 | 0.6849 |
|  | Age | 0.0626 | 0.1813 | 0.35 | 0.7308 |
|  | BMI | -0.4844 | 0.3774 | -1.28 | 0.2032 |

* indicates significant difference at *p*<0.05. ALT, alanine aminotransferase; POD, postoperative day; NRS, numeric rating scale; BMI, body mass index; PAI, peri-articular injection; IAI, intra-articular injection.

**Supplementary Table 5**

Contribution to the change in CK from before surgery to POD 1 and POD 7.

|  | Dependent variables | Estimate | Standard error | *T* ratio | *p*-value |
| --- | --- | --- | --- | --- | --- |
| Change from before surgery to POD 1 | |  |  |  |  |
| Control group (*N*=113) | |  |  |  |  |
|  | Intercept | 667.6686 | 267.7210 | 2.49 | 0.0142* |
|  | Preoperative NRS scores | -34.0500 | 13.1067 | -2.60 | 0.0107* |
|  | Sex (female) | 41.8215 | 36.8251 | 1.14 | 0.2586 |
|  | Age | 0.0684 | 2.6400 | 0.03 | 0.9794 |
|  | BMI | -6.7058 | 8.4229 | -0.80 | 0.4277 |
| PAI group (*N*=87) | |  |  |  |  |
|  | Intercept | 236.8568 | 155.3109 | 1.53 | 0.1311 |
|  | Preoperative NRS scores | -11.1905 | 7.5097 | -1.49 | 0.1400 |
|  | Sex (female) | -28.7141 | 24.1537 | -1.19 | 0.2379 |
|  | Age | 0.3240 | 1.7670 | 0.18 | 0.8550 |
|  | BMI | 1.6282 | 3.1876 | 0.51 | 0.6109 |
| IAI group (*N*=81) | |  |  |  |  |
|  | Intercept | 477.4047 | 127.6825 | 3.74 | 0.0004* |
|  | Preoperative NRS scores | -11.4620 | 6.9113 | -1.66 | 0.1013 |
|  | Sex (female) | -25.7040 | 24.5206 | -1.05 | 0.2978 |
|  | Age | -1.7099 | 1.4775 | -1.16 | 0.2508 |
|  | BMI | -1.9548 | 3.0758 | -0.64 | 0.5270 |
| Change from before surgery to POD 7 | |  |  |  |  |
| Control group (*N*=113) | |  |  |  |  |
|  | Intercept | 114.4174 | 62.5684 | 1.83 | 0.0702 |
|  | Preoperative NRS scores | -3.6020 | 3.0631 | -1.18 | 0.2422 |
|  | Sex (female) | -3.7096 | 8.6063 | -0.43 | 0.6673 |
|  | Age | -0.5285 | 0.6170 | -0.86 | 0.3936 |
|  | BMI | -2.4503 | 1.9685 | -1.24 | 0.2159 |
| PAI group (*N*=87) | |  |  |  |  |
|  | Intercept | 236.8568 | 155.3109 | 1.53 | 0.1311 |
|  | Preoperative NRS scores | -11.1905 | 7.5097 | -1.49 | 0.1400 |
|  | Sex (female) | -28.7141 | 24.1537 | -1.19 | 0.2379 |
|  | Age | 0.3240 | 0.17670 | 0.18 | 0.8550 |
|  | BMI | 1.6282 | 3.1877 | 0.51 | 0.6109 |
| IAI group (*N*=81) | |  |  |  |  |
|  | Intercept | 477.4047 | 127.6825 | 3.74 | 0.0004* |
|  | Preoperative NRS scores | -11.4620 | 6.9113 | -1.66 | 0.1013 |
|  | Sex (female) | -25.7040 | 24.5206 | -1.05 | 0.2978 |
|  | Age | -1.7099 | 1.4775 | -1.16 | 0.2508 |
|  | BMI | -1.9548 | 3.0758 | -0.64 | 0.5270 |

* indicates significant difference at *p*<0.05. CK, creatine phosphokinase; POD, postoperative day; NRS, numeric rating scale; BMI, body mass index; PAI, peri-articular injection; IAI, intra-articular injection.

**Supplementary Table 6**

Contribution to the change in BUN from before surgery to POD 1 and POD 7.

|  | Dependent variables | Estimate | Standard error | *T* ratio | *p*-value |
| --- | --- | --- | --- | --- | --- |
| Change from before surgery to POD 1 | |  |  |  |  |
| Control group (*N*=113) | |  |  |  |  |
|  | Intercept | -141.4689 | 805.762 | -0.18 | 0.8610 |
|  | Preoperative NRS scores | 64.9275 | 39.4473 | 1.65 | 0.1027 |
|  | Sex (female) | 25.0675 | 110.8328 | 0.23 | 0.8215 |
|  | Age | 5.9057 | 7.9457 | 0.74 | 0.4589 |
|  | BMI | -22.4811 | 25.3505 | -0.89 | 0.3772 |
| PAI group (*N*=87) | |  |  |  |  |
|  | Intercept | -2.0051 | 4.1764 | -0.48 | 0.6324 |
|  | Preoperative NRS scores | 0.1245 | 0.2019 | 0.62 | 0.5391 |
|  | Sex (female) | -0.1847 | 0.6495 | -0.28 | 0.7768 |
|  | Age | -0.0124 | 0.0475 | -0.26 | 0.7948 |
|  | BMI | -0.0497 | 0.0857 | -0.58 | 0.5639 |
| IAI group (*N*=81) | |  |  |  |  |
|  | Intercept | -2.0051 | 4.1763 | -0.48 | 0.6324 |
|  | Preoperative NRS scores | 0.1245 | 0.2019 | 0.62 | 0.5391 |
|  | Sex (female) | -0.1847 | 0.6495 | -0.28 | 0.7768 |
|  | Age | -0.0124 | 0.0475 | -0.26 | 0.7948 |
|  | BMI | -0.0497 | 0.0857 | -0.58 | 05639 |
| Change from before surgery to POD 7 | |  |  |  |  |
| Control group (*N*=113) | |  |  |  |  |
|  | Intercept | -1.7429 | 3.7753 | -0.46 | 0.6454 |
|  | Preoperative NRS scores | -0.0543 | 0.1848 | -0.29 | 0.7695 |
|  | Sex (female) | -1.3548 | 0.5193 | -2.61 | 0.0104* |
|  | Age | 0.0145 | 0.0372 | 0.40 | 0.6905 |
|  | BMI | -0.0124 | 0.1188 | -0.10 | 0.9168 |
| PAI group (*N*=87) | |  |  |  |  |
|  | Intercept | -2.8376 | 4.6001 | -0.62 | 0.5390 |
|  | Preoperative NRS scores | -0.0623 | 0.2224 | -0.28 | 0.7802 |
|  | Sex (female) | 0.0741 | 0.7154 | 0.10 | 0.9177 |
|  | Age | 0.0177 | 0.0523 | 0.34 | 0.7359 |
|  | BMI | 0.1223 | 0.0944 | 1.30 | 0.1988 |
| IAI group (*N*=81) | |  |  |  |  |
|  | Intercept | -5.2851 | 3.5940 | -1.47 | 0.1456 |
|  | Preoperative NRS scores | -0.0199 | 0.1945 | -0.10 | 0.9190 |
|  | Sex (female) | -1.8200 | 0.6902 | -2.64 | 0.0101* |
|  | Age | 0.1362 | 0.0416 | 3.27 | 0.0016* |
|  | BMI | 0.0151 | 0.0866 | 0.17 | 0.8614 |

* indicates significant difference at *p*<0.05. BUN, blood urea nitrogen; POD, postoperative day; NRS, numeric rating scale; BMI, body mass index; PAI, peri-articular injection; IAI, intra-articular injection.

**Supplementary Table 7**

Contribution to the change in Cr from before surgery to POD 1 and POD 7.

|  | Dependent variables | Estimate | Standard error | *T* ratio | *p*-value |
| --- | --- | --- | --- | --- | --- |
| Change from before surgery to POD 1 | |  |  |  |  |
| Control group (*N*=113) | |  |  |  |  |
|  | Intercept | -0.1102 | 0.0570 | -1.93 | 0.0560 |
|  | Preoperative NRS scores | -0.0017 | 0.0028 | -0.61 | 0.5454 |
|  | Sex (female) | 0.0034 | 0.0078 | 0.43 | 0.6690 |
|  | Age | 0.0005 | 0.0006 | 0.93 | 0.3551 |
|  | BMI | 0.0004 | 0.0018 | 0.22 | 0.8279 |
| PAI group (*N*=87) | |  |  |  |  |
|  | Intercept | -0.0444 | 0.0568 | -0.78 | 0.4364 |
|  | Preoperative NRS scores | -0.0011 | 0.0027 | -0.40 | 0.6901 |
|  | Sex (female) | 0.0051 | 0.0088 | 0.57 | 0.5684 |
|  | Age | 0.00004 | 0.0006 | 0.06 | 0.9526 |
|  | BMI | -0.0011 | 0.0012 | -0.96 | 0.3381 |
| IAI group (*N*=81) | |  |  |  |  |
|  | Intercept | -0.1568 | 0.0606 | -2.59 | 0.0115* |
|  | Preoperative NRS scores | 0.0013 | 0.0033 | 0.40 | 0.6881 |
|  | Sex (female) | -0.0161 | 0.0116 | -1.39 | 0.1700 |
|  | Age | 0.0006 | 0.0007 | 0.84 | 0.4044 |
|  | BMI | 0.0020 | 0.0015 | 1.37 | 0.1760 |
| Change from before surgery to POD 7 | |  |  |  |  |
| Control group (*N*=113) | |  |  |  |  |
|  | Intercept | -0.0759 | 0.0587 | -1.29 | 0.1981 |
|  | Preoperative NRS scores | -0.0022 | 0.0029 | -0.78 | 0.4395 |
|  | Sex (female) | 0.0075 | 0.0081 | 0.92 | 0.3572 |
|  | Age | -0.0002 | 0.0006 | -0.28 | 0.7834 |
|  | BMI | 0.0030 | 0.0018 | 1.65 | 0.1027 |
| PAI group (*N*=87) | |  |  |  |  |
|  | Intercept | -0.0862 | 0.1355 | -0.64 | 0.5266 |
|  | Preoperative NRS scores | 0.0137 | 0.0066 | 2.08 | 0.0406* |
|  | Sex (female) | 0.0020 | 0.0211 | 0.09 | 0.9248 |
|  | Age | -0.0012 | 0.0015 | -0.75 | 0.4526 |
|  | BMI | 0.0026 | 0.0028 | 0.94 | 0.3492 |
| IAI group (*N*=81) | |  |  |  |  |
|  | Intercept | -0.2161 | 0.0705 | -3.07 | 0.0030* |
|  | Preoperative NRS scores | 0.0015 | 0.0038 | 0.38 | 0.7044 |
|  | Sex (female) | 0.0034 | 0.0135 | 0.25 | 0.8017 |
|  | Age | 0.0009 | 0.0008 | 1.06 | 0.2927 |
|  | BMI | 0.0050 | 0.0017 | 2.93 | 0.0044* |

* indicates significant difference at *p*<0.05. Cr, creatinine; POD, postoperative day; NRS, numeric rating scale; BMI, body mass index; PAI, peri-articular injection; IAI, intra-articular injection.

**Supplementary Table 8**

Contribution to the change in CRP from before surgery to POD 1 and POD 7.

|  | Dependent variables | Estimate | Standard error | *T* ratio | *p*-value |
| --- | --- | --- | --- | --- | --- |
| Change from before surgery to POD 1 | |  |  |  |  |
| Control group (*N*=113) | |  |  |  |  |
|  | Intercept | 0.4653 | 1.8549 | 0.25 | 0.8024 |
|  | Preoperative NRS scores | 0.0995 | 0.0908 | 1.10 | 0.2757 |
|  | Sex (female) | -0.0650 | 0.2551 | -0.25 | 0.7993 |
|  | Age | 0.0435 | 0.0183 | 2.38 | 0.0191* |
|  | BMI | 0.0433 | 0.0584 | 0.74 | 0.4596 |
| PAI group (*N*=87) | |  |  |  |  |
|  | Intercept | 1.7628 | 1.6000 | 1.10 | 0.2738 |
|  | Preoperative NRS scores | -0.0864 | 0.0774 | -1.12 | 0.2675 |
|  | Sex (female) | 0.0840 | 0.2488 | 0.34 | 0.7365 |
|  | Age | 0.0178 | 0.0182 | 0.98 | 0.3304 |
|  | BMI | 0.0175 | 0.0328 | 0.53 | 0.5966 |
| IAI group (*N*=81) | |  |  |  |  |
|  | Intercept | -1.4714 | 1.6078 | -0.92 | 0.3630 |
|  | Preoperative NRS scores | 0.0491 | 0.0870 | 0.56 | 0.5742 |
|  | Sex (female) | 0.5268 | 0.3088 | 1.71 | 0.0921 |
|  | Age | 0.0413 | 0.0186 | 2.22 | 0.0296* |
|  | BMI | 0.0191 | 0.0387 | 0.49 | 0.6241 |
| Change from before surgery to POD 7 | |  |  |  |  |
| Control group (*N*=113) | |  |  |  |  |
|  | Intercept | 3.0669 | 1.5537 | 1.97 | 0.0510 |
|  | Preoperative NRS scores | 0.0297 | 0.0761 | 0.39 | 0.6973 |
|  | Sex (female) | -0.2831 | 0.2137 | -1.32 | 0.1881 |
|  | Age | 0.0017 | 0.0153 | 0.11 | 0.9119 |
|  | BMI | -0.0448 | 0.0488 | -0.92 | 0.3611 |
| PAI group (*N*=87) | |  |  |  |  |
|  | Intercept | -0.3292 | 1.1698 | -0.28 | 0.7791 |
|  | Preoperative NRS scores | 0.0445 | 0.0566 | 0.79 | 0.4338 |
|  | Sex (female) | 0.0264 | 0.1819 | 0.14 | 0.8851 |
|  | Age | 0.0034 | 0.0133 | 0.26 | 0.7971 |
|  | BMI | 0.01143 | 0.0240 | 0.48 | 0.6354 |
| IAI group (*N*=81) | |  |  |  |  |
|  | Intercept | -0.1796 | 0.4952 | -0.36 | 0.7178 |
|  | Preoperative NRS scores | -0.0112 | 0.0268 | -0.42 | 0.6761 |
|  | Sex (female) | 0.0514 | 0.0951 | 0.54 | 0.5907 |
|  | Age | 0.0100 | 0.0057 | 1.74 | 0.0853 |
|  | BMI | -0.0052 | 0.0119 | -0.44 | 0.6621 |

* indicates significant difference at *p*<0.05. CRP, C-reactive protein; POD, postoperative day; NRS, numeric rating scale; BMI, body mass index; PAI, peri-articular injection; IAI, intra-articular injection.

**Supplementary Table 9**

Contribution to the change in D-dimer from before surgery to POD 1 and POD 7.

|  | Dependent variables | Estimate | Standard error | *T* ratio | *p*-value |
| --- | --- | --- | --- | --- | --- |
| Change from before surgery to POD 1 | |  |  |  |  |
| Control group (*N*=113) | |  |  |  |  |
|  | Intercept | 5.4431 | 2.3904 | 2.28 | 0.0248* |
|  | Preoperative NRS scores | 0.0571 | 0.1170 | 0.49 | 0.6264 |
|  | Sex (female) | 0.3810 | 0.3288 | 1.16 | 0.2491 |
|  | Age | -0.0052 | 0.0236 | -0.22 | 0.8256 |
|  | BMI | -0.1369 | 0.0752 | -1.82 | 0.0715 |
| PAI group (*N*=87) | |  |  |  |  |
|  | Intercept | 6.5981 | 2.8085 | 2.35 | 0.0212* |
|  | Preoperative NRS scores | 0.0372 | 0.1358 | 0.27 | 0.7851 |
|  | Sex (female) | -0.1638 | 0.4368 | -0.37 | 0.7087 |
|  | Age | -0.0177 | 0.0320 | -0.55 | 0.5804 |
|  | BMI | -0.1334 | 0.0576 | -2.31 | 0.0232 |
| IAI group (*N*=81) | |  |  |  |  |
|  | Intercept | 2.9003 | 2.9813 | 0.97 | 0.3337 |
|  | Preoperative NRS scores | 0.1149 | 0.1614 | 0.71 | 0.4788 |
|  | Sex (female) | -0.7843 | 0.5725 | -1.37 | 0.1748 |
|  | Age | 0.0295 | 0.0344 | 0.86 | 0.3952 |
|  | BMI | -0.0866 | 0.0718 | -1.21 | 0.2315 |
| Change from before surgery to POD 7 | |  |  |  |  |
| Control group (*N*=113) | |  |  |  |  |
|  | Intercept | 8.3786 | 1.8770 | 4.46 | <0.0001* |
|  | Preoperative NRS scores | -0.1750 | 0.0919 | -1.90 | 00595 |
|  | Sex (female) | 0.5944 | 0.2582 | 2.30 | 0.0232* |
|  | Age | -0.0063 | 0.0185 | -0.34 | 0.7321 |
|  | BMI | -0.0935 | 0.0591 | -1.58 | 0.1162 |
| PAI group (*N*=87) | |  |  |  |  |
|  | Intercept | 5.0034 | 1.9852 | 2.52 | 0.0137* |
|  | Preoperative NRS scores | -0.1035 | 0.0960 | -1.08 | 0.2842 |
|  | Sex (female) | -0.1587 | 0.3087 | -0.51 | 0.6087 |
|  | Age | 0.0027 | 0.0226 | 0.12 | 0.9045 |
|  | BMI | -0.0646 | 0.0407 | -1.59 | 0.1165 |
| IAI group (*N*=81) | |  |  |  |  |
|  | Intercept | 1.7243 | 1.2227 | 1.41 | 0.1626 |
|  | Preoperative NRS scores | 0.0257 | 0.0662 | 0.39 | 0.6990 |
|  | Sex (female) | 0.2543 | 0.2348 | 1.08 | 0.2823 |
|  | Age | 0.0202 | 0.0141 | 1.43 | 0.1572 |
|  | BMI | -0.0380 | 0.0295 | -1.29 | 0.2006 |

* indicates significant difference at *p*<0.05. POD, postoperative day; NRS, numeric rating scale; BMI, body mass index; PAI, peri-articular injection; IAI, intra-articular injection.
